# Spectroscopic and Electrochemical Analysis of Psychotropic Drugs

**DOI:** 10.4103/0250-474X.51942

**Published:** 2009

**Authors:** H. Puzanowska-Tarasiewicz, W. Misiuk, K. Mielech-Łukasiewicz, L. Kuźmicka

**Affiliations:** Institute of Chemistry, University of Białystok, Hurtowa 1, Białystok 15-399, Poland

**Keywords:** Psychotropic drugs, spectrophotometric and electrochemical methods, analysis

## Abstract

Psychotropic drugs are an important family of compounds from a medical point of view. Their application in therapy requires methods for the determination in pharmaceutical dosage forms and body fluids. Several methods for their analysis have been reported in the literature. Among the methods, spectrophotometric and electrochemical are very useful for the determination of the drugs. Some of the spectrophotometric methods are based on the formation of the binary and ternary compounds with complexes of metals. The formed compounds are sparingly soluble in water, but quantitatively extracted from aqueous phase into organic solvents and the extracts are intensely colored and stable for a few days. These complexes have been employed in pharmaceutical analysis. The electrochemical procedures are very useful in determination of the psychotropic substances in pharmaceutical preparations.

Psychotropic drugs belong to a large group of organic compounds. They exhibit high activity and many-sided pharmacological actions. Owing to these properties, they have been subject of extensive pharmacological studies. Typical psychotropic drugs are often prescribed in severe cases of schizophrenia or depression, when new generation neuroleptics and SSRI medications do not work. Typical tricyclic psychotropic drugs are characterized by a tricyclic rings and presence of sulfur and nitrogen atoms. Structures of select psychotropic drugs of both typical and atypical type are presented in fig. 1.

**Fig. 1:**
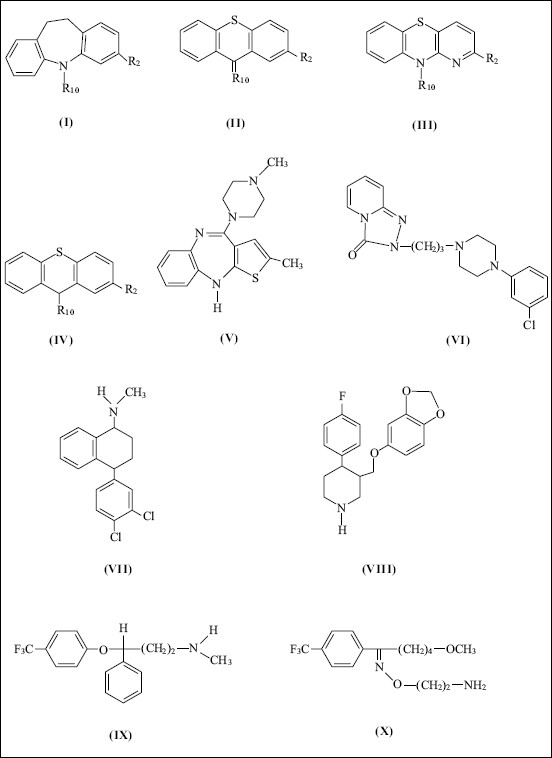
Formulae of antipsychotic drugs Formulae of dibenzoazepines (I), thioxanthenes (II), azaphenothiazines (III) phenothiazines (IV), olanzapine (V), trazodone (VI), sertraline (VII), paroxetine (VIII), fluoxetine (IX), fluvoxamine (X)

Tricyclic psychotropic drugs due to their characteristic structure – the presence of chemically active sulfur and nitrogen atoms and substituents react with oxidants (e.g. Os(VIII), Cr(VI), V(V), Ce(IV), Au(III), Fe(III)), platinum metals (e.g. Pt(IV), Rh(IV), Ru(III), Pd(II)), thiocyanate or halide complexes of metals and some organic substances (e.g. picric acid, alizarin S, dipicrylamine)[1–6]. The above mentioned reactions are very important from an analytical point of view.

The liability of phenothiazines, thioxantenes and dibenzoazepines to the oxidation in acid medium by K_2_Cr_2_O_7_, NH_4_VO_3_, Ce(SO_4_)_2_ has been exploited as indicators in various redox titrations[5,7] and as reagents for the spectrophotometric determination of the drugs[8–10].

The new generation psychotropic drugs, e.g., fluoxetine, fluvoxamine and trazodone react with some organic compounds such as chrome azurol S, eriochrome cyanine R, bromophenol blue, methyl orange, bromocresol green and thymol blue to form ion-association compounds[11–16]. These properties have been exploited for the development of spectrophotometric methods for the determination of the psychotropic drugs.

Atypical psychotropic drugs can also easily reduced on mercury electrode[17,18]. Mechanism of electrochemical reactions of these compounds was investigated using different electrochemical methods, e.g. cyclic voltammetry and differential pulse voltammetry[19]. Electrochemical behaviour of these substances can be successfully employed for elaborate simple, rapid and sensitive procedures for the determination of new psychotropic drugs in pharmaceutical preparations and biological fluids.

In the presented review the analytical applications of the reactions of psychotropic drugs with organic substances and thiocyanate complexes of metals for the spectrophotometric determination of the drugs have been described. This review is also devoted to the analytical application of the electrochemical methods for their determination.

## Binary and ternary complexes of psychotropic drugs:

Psychotropic substances, e.g. phenothiazines, dibenzocycloheptadienes and thioxantenes react with organic substances, which occur as anions in aqueous solutions forming colored ion-association binary compounds[20]. These compounds are insoluble into organic solvents, e.g. chloroform, butanol and benzene. The extracts are intensely colored and very stable (1-3 days)[21]. These properties were applied successfully for the spectrophotometric determination of psychotropic drugs[21–41] (Table 1).

**TABLE 1:** DETERMINATION OF PSYCHOTROPIC DRUGS IN BINARY SYSTEMS

Psychotropic drugs	Organic reagents	Organic solvent	λ [nm]	ε [l·^−^mol^−1^cm^−1^]	Range of determination [ppm]	Ref.
Perphenazine	Dipicrylamine	chloroform	420	1.09·10^4^	8 - 60	21
Chlorpromazine		chloroform	420	3.22·104	0.7 - 7	22
Thioproperazine			435	1.51·10^4^	1.6-16	
Chlorpromazine	Alizarin S	chloroform	420	8.00·10^3^	7 - 70	23
Promethazine		chloroform	420	8.50·10^3^	7 - 70	24
Chlorpromazine	Brilliant blue	chloroform	620	2.21·10^4^	1 - 10	25
Fluphenazine				1.02·10^4^	2 - 10	
Thioridazine				2.78·10^4^	1 - 10	
Levomepromazine	Bromophenol blue	chloroform	409	-	5 - 25	26
Fluoxetine	Chrome	chloroform	500	1.02·10^4^	5-50	12
Fluvoxamine	Azurol S	chloroform-butanol (3:1)	502	9.05·10^3^	7-100	
Chlorpromazine		chloroform	510	1.48·10^4^	2 - 20	27
Promethazine				2.04·10^4^	1 - 12	
Thioproperazine			460	1.57·10^4^	2 - 28	28
Trifluopromazine				2.12·10^4^	1 - 12	
Promethazine	Methyl orange	chloroform	-	-	20-100	29
Imipramine	Eriochrome Cyanine R	butanol	520	4.80·10^3^	10 - 80	30
Chlorpromazine	Pyrocatechol	chloroform -	445	1.04·10^4^	3.5 - 35	31
Chlorprothixene	violet	butanol (5:1)				
		chloroform - butanol (5:1)	445	1.40·10^4^	3.5 - 32	32
Chlorpromazine	Flavianic acid	benzene	390	9.60·10^3^	7- 70	33
Thioridazine			385	-	5 - 20	34
Promazine	Picramic acid	chloroform	500	2.10·10^3^	8 - 80	35
Thioproperazine				-	16 - 160	36
Thioridazine	Picric acid	benzene	405	-	20 - 70	34
Thioproperazine			406	7.30·10^3^	10 - 80	33
Trifluoperazine			406	6.70·10^3^	10 - 100	37
Perphenazine			407	7.60·10^3^	4-80	21
Promazine			405	-	10-60	38
Methopromazine						
Promethazine	Orange II	dichloro-methane	485	-	5 - 20	25
Fluphenazine				1.02·10^4^	3 - 25	
Prochlorpromazine		chloroform	495	5.40·10^4^	30-130	
Trifluoperazine				1.14·10^4^	3 - 25	39
Nortriptyline		chloroform	490	-	-	
Chlorpromazine	Bromocresol green	chloroform	420	2.63·10^4^	2 - 8	40
Trifluorpromazine				2.02·10^4^	2 -10	
Thioproperazine				2.65·10^4^	2 -12	
Thioridazine				2.13·10^4^	2 -18	
Chlorpromazine	Titanium yellow	ethyl acetate	405	-	10 - 60	41
Fluoxetine	Eriochrome	butanol	520	1.7·10^4^	2-30	13
Fluvoxamine	cyanine R		518	6.5·10^3^	2-40	
Trazodone	Bromophenol blue	chloroform	414	-	3.75-14	11

Line denotes for lack reference

Significant advantages of the spectrophotometric methods are that they can be applied to the determination of individual components in a multicomponent mixture. This aspect of spectrophotometric analysis is of major interest in pharmacy, since it offers distinct possibilities in the assay of a particular component in a complex dosage formulation. For example in the spectrophotometric method elaborated by Basavaiah[40], the commonly used additives and excipients in the dosage forms of active compounds, such as starch, lactose, glucose, sugar, talc, gelatin, magnesium stearate, sodium lauryl sulphate, sodium sulphite, sodium chloride, calcium chloride, ethanol, formaldehyde and sodium salt of EDTA did not interfere in the analysis.

It has been pointed out in our previous papers[2,4,42] that active substances of psychotropic drugs (PS), which occur in an aqueous solution as large cation, PS·HCl⇄(PS·H)^+^+Cl^−^, or base, PS+H^+^⇄(PS·H)+, react with some thiocyanate and halide complexes of metals forming ion-association compounds, (m-n)(PS·H)^+^+[MeXn](m-n)^−^⇄(PS·H)(m-n)[MeXn], where, Me denotes metal ion with an n-oxidation state (*e.g.* Co(II), Cd(II), Hg(II), Pd(II), Fe(III), Cr(III), Ti(IV), Pt(IV), Re(IV), Nb(V), Mo(V), W(V), U(VI)); X- SCN^-^ or halide ion. These compounds exhibit a number of properties very important from the analytical view-point, i.e, a well-defined composition and high molecular weight. They are hardly soluble in water but fairly soluble in acetone, methanol, ethanol. They can be extracted from aqueous phase with chloroform and other organic solvents. The extracts are intensely colored and stable for a few days[2]. These properties have been used for the development of the spectrophotometric methods for the determination of psychotropic drugs.

## Structures of binary and ternary complexes of psychotropic drugs:

**Scheme 1:**
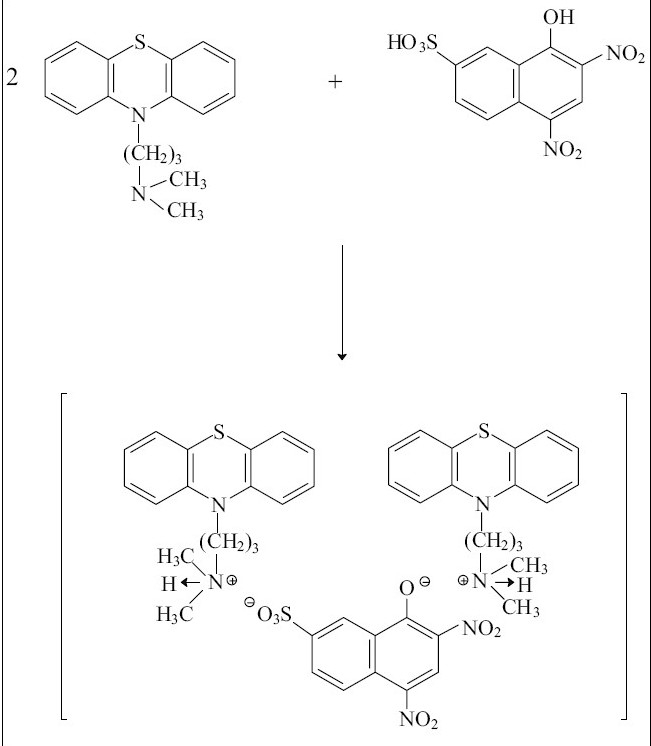
Possible structure of promazine-flavianic acid compound

**Scheme 2:**
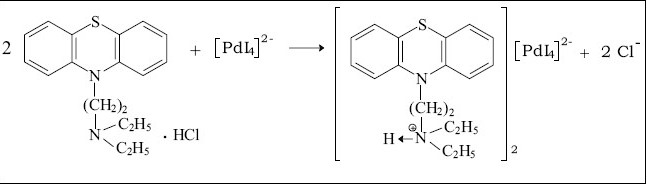
The reaction of diethazine hydrochloride with [PdI_4_]^2−^ The reaction of the protonated amine nitrogen of diethazine hydrochloride with the anionic complex of [PdI_4_]^2−^

The compositions of binary and ternary complexes of psychotropic drugs, e.g., dibenzoazepines, dibenzocycloheptadienes, thioxanthenes, phenothiazines were established by Job's continuous variation method and by spectrophotometric titration. These compositions are described in the literature[2,4,43,44]. The absorption spectra of the compounds obtained in the UV/VIS and IR-region have been recorded. It was found the main absorption bands of the components are observed in UV-VIS spectra of the compounds[2,43,45]. Infrared spectra of the compounds studied were recorded (KBr_disc_) in the region of 400-4000 cm^−1^[2,27,45,46]. Significant changes in the spectra were observed in the region of 2300-3700 cm^−1^. For example, the wide bands, appearing in the phenothiazines spectra in the region of 2300-2700 cm^−1^ and characteristic for vibration of ≡NH^+^ group, were shifted (~350 cm^−1^) towards higher frequencies in the spectra of the compounds. On the basis of the data obtained, it has been established that the compounds studied are ion-associates. For example, the structure of the compound promazine with flavianic acid and compound obtained in Pd(II) - I^−^ - DT system can be presented as follows (Schemes 1 and 2).

## Spectrophotometric methods:

Reviews of the methods for the determination of phenothiazines presented by Blažek[47], Fairbrother[48] and Puzanowska-Tarasiewicz[49] show that spectrophotometric methods are very useful for the determination of psychotropic substances in pharmaceuticals and body fluids[50–56]. They are summarized in Table 2.

**TABLE 2:** DETRMINATION OF PSYCHOTROPIC DRUGS IN TERNARY SYSTEMS

Psychotropic substance	System	Organic solvent	λ [nm]	ε [l·mol^−1^cm^−1^]	Range of determination [ppm]	Ref.
Levomepromazine (LPZ)	LPZ-[Cr(NH_3_)_2_(SCN)_4_]	acetone	525	9.12·10^4^	53 - 427	45
Chlorpromazine (CPZ)	CPZ-[Cr(NH_3_)_2_(SCN)_4_]	acetone	520	9.12·10^4^	156 - 689	50
Chlorpromazine (CPZ)	Fe(III)-SCN^-^-CPZ	chloroform	490	-	120 - 300	51
Levomepromazine (LPZ)	Fe(III)-SCN^-^-LPZ				140 - 400	
Promethazine (PMT)	Fe(III)-SCN^-^-PMT				160 - 550	
Chlorpromazine (CPZ)	Co(II)-SCN^-^-CPZ	ether	620	-	100 - 600	52
Chloracizine (CRZ)	Co(II)-SCN^-^-CRZ				100 - 900	
Chlorpromazine (CPZ)	Ge(IV)-PCV-CPZ	cyclohexa-none	580	6.8 ·10^3^	7 - 70	53
Chlorpromazine (CPZ)	Sn(IV)-PCV-CPZ	butanol	580	-	2 - 20	31
Desipramine (DE)	Ti(IV)- SCN^-^- DE	chloroform	355	5.85 ·10^4^	5 - 200	54
Thioridazine (TR)	Ti(IV) - SCN^-^- TR	chloroform -	417	-	20 - 160	
Perazine (PZ)	Ti(IV) - SCN^-^-PZ	butanol (4:1)	360	8.96·10^3^	20 - 170	55
Amitriptyline (AM)	Ti(IV) - SCN^-^-AM				3 - 60	
Promazine (PM)	Nb(V)-SCN^-^-PM	trichloro-ethylene	400	-	20 - 200	56
Imipramine (IM)	Nb(V)- SCN^-^- IM	butanol-chloroform (1:9)	350	6.67·10^4^	0.8 - 8	43
Doxepin (DX)	Ti(IV)- SCN^-^- DX	butanol-chloroform (2:3)	400	7.12· 10^3^	5 - 50	44
Chlorprothixene (CX)	Nb(V)- SCN^-^- CX	butanol	362	8·10^3^	9 - 50	46

Line denotes for lack reference

Recently Misiuk[44] studied the ion association compounds of doxepin (DX) with thiocyanate complexes of titanium (IV) and iron (III). The produced compounds were insoluble in water, but well soluble in some organic solvents. They were quantitatively extracted with a mixture of butanol-chloroform (2:3) and (1:4) using titanium (IV) and iron (III) thiocyanates, respectively. The mentioned properties were applied for the elaboration of new spectrophotometric methods for the determination of doxepin in DX-Ti-SCN^-^ and DX-Fe-SCN^-^ systems, respectively. The proposed methods have been successfully applied for the determination of the main active ingredient in different dosage forms. The method can also be used for the determination of doxepin in the presence of its degradation product, dibenzo[b,e]oxepin-11-(6H)-one. The dibenzo[b,e] oxepin-11-(6H)–one was examined by TLC, UV and IR techniques. The precision, accuracy and reproducibility of the methods were good and RSD values were low.

It has been found that imipramine and chlorprothixene react with thiocyanate complexes of niobium (V) forming yellow sparingly soluble in water compound in a molar ratio of 1:2 of Nb (V):each drug[43,46]. These compounds can be quantitatively extracted with chloroform-butanol (1:9) or butanol alone. The spectrophotometric methods have been developed for the determination of imipramine and chlorprothixene in the ranges of 0.8-8 ppm and 9-50 ppm, respectively.

Desipramine forms a compound of the ion pair type with thiocyanate complexes of titanium (IV)[54] in acid medium, which can be quantitatively extracted with chloroform. The properties have been used for the spectrophotometric determination of desipramine in the range of 5 - 200 ppm.

Chlorpromazine (CPZ) forms a yellow sparingly soluble in water compound of molar ratio of 1:1 (λ_max_ = 445 nm) with pyrocatechol violet (PCV) in an acid medium[31]. The drug also reacts with tin(IV) ions in the presence of pyrocatechol violet, in aqueous phase at the molar ratio Sn(IV):PCV:CPZ = 1:2:2, and in the organic phase at the ratio 1:2:4 (λ_max_ = 580 nm). These compounds can be quantitatively extracted from aqueous solutions with chloroform-butanol (5:1) or butanol alone. Taking advantage of these properties, the spectrophotometric methods for the determination of chlorpromazine have been developed. The methods proved suitable for assaying chlorpromazine in the range of its concentrations from 3.5 ppm to 35 ppm and from 2 ppm to 20 ppm, respectively. The reaction in the systems chlorpromazine-chrome azurol S[27] and germanium (IV)-pyrocatechol violet-chlorpromazine[53] were applied for CPZ determination in the concentration range 2-20 ppm and 7-70 ppm, respectively.

**Scheme 3:**
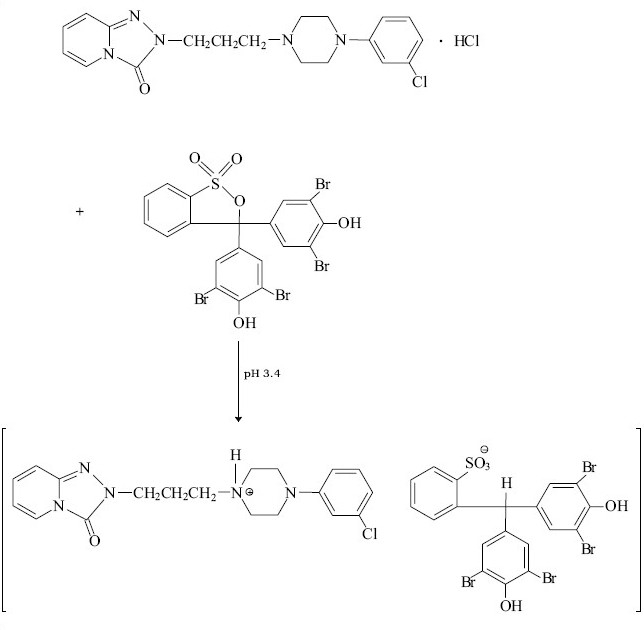
Mechanism of ion pair complex trazodone-bromophenol blue system

Fluoxetine and fluvoxamine were determined spectrophotometrically using chrome azurol S and eriochrome cyanine R[12,13]. These dyes react in aqueous media with studied psychotropic drugs forming colored, sparingly soluble in water complexes. These complexes can be quantitatively extracted with chloroform or chloroform-buthanol (3:1). Gindy *et al.*[11] described the methods for the determination of trazodone in pharmaceutical preparations. The spectrophotometric and spectrofluorimetric methods were based on the formation of yellow ion pair complex between the basic nitrogen of the drug and bromophenol blue. The formed complex was extracted with chloroform and the absorbance was measured at 414 nm. The suggested mechanism of trazodone – bromophenol blue ion pair complex is presented in Scheme 3.

## Electrochemical methods:

Psychotropic active substances are easily oxidized (e.g., dibenzoazepines, thioxanthenes, phenothiazines) or reduced (e.g., trazodone, sertraline, paroxetine), electrochemically. The first step in the electrochemical oxidation of phenothiazine and azaphenothiazine derivatives occurs at the sulphur atom, while the second wave is attributed to the transformation of the radical cation into a dication[5]. The mechanism of the oxidation of thioxanthenes is not fully understood, but the potentials and peaks shapes of the thioxanthene derivatives are similar to those of the phenothiazine derivatives, the first oxidation wave involving two-electron oxidation to the sulphoxide[1]. The dibenzoazepines are the most easily oxidized, the first electron is removed from the monomer nitrogen and the radical can then exist in a number of resonance forms. The monocation rapidly dimerises or reacts with an unoxidised molecule. The dimerisation is accompanied by the loss of two protons per dimer. The dimer is more easily oxidized than the monomer[57].

Electrochemical oxidation of some active substances is exploited for using these substances in polarographic and voltammetric analysis. Several electrochemical techniques have been applied for the determination of psychotropic substances in drugs preparations and differential biological samples[58–60]. Oelschlager[59] reviewed the polarographic methods reported for psychotropic drugs including phenothiazine and azaphenothiazine derivatives. Temsamani *et al.*[61] have analyzed chlorpromazine in plasma by using cyclic voltammetry and modified gold electrode. Chlorpromazine has been determined in urine samples of patients by adsorptive stripping voltammetry in the presence of Triton X-100[62]. Fluphenazine and trifluoperazine were determined by differential pulse voltammetry after pre-concentration at a wax-impregnated graphite electrode. For plasma, the electrode was covered with a membrane to prevent fouling by proteins[63]. Alternatively, promazine, chlorpromazine and promethazine spiked in urine samples were oxidized by nitrous acid into the corresponding sulphoxides, which were polarographically active. They produce well-defined diffusion-controlled cathodic wave[58].

The dibenzoazepine derivatives, e.g. imipramine, clomipramine and trimipramine were determined in drugs preparations and plasma samples with adsorptive stripping voltammetry[64,65]. Imipramine and desipramine were analysed by cyclic voltammetry at glassy carbon and boron-doped diamond electrode[66]. Wang *et al.*[67] determined imipramine and trimipramine in urine sample by using cyclic voltammetry and differential pulse voltammetry. Among thioxanthene derivatives, zuclopenthixol was determined quantitatively by measuring the height of voltammetric peaks. The oxidative voltammetric behaviour of zuclopenthixol at a glassy carbon electrode has been studied using cyclic, linear sweep and differential pulse voltammetry[68]. Tuzhi *et al.*[69] reported an adsorptive pre-concentration method for the voltammetric measurement of trace levels of chlorprothixene. Alternatively, chlorprothixene and thiothixene were determined polarographically through the formation of their bromo-derivatives, which manifest well defined cathodic waves in select supporting electrolytes[70].

**Fig. 2:**
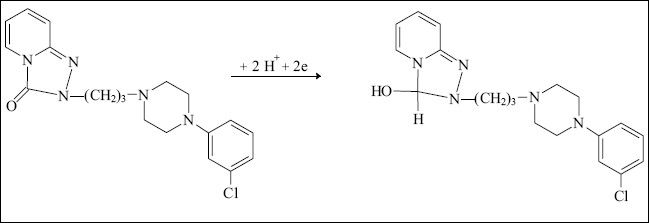
Mechanism of electroreduction of trazodone

Trazodone, which is a triazolopyridine derivative, unlike the tricyclic antidepressants was studied using direct current, differential pulse and alternating current polarography[71]. It was concluded that, only the carbonyl group was involved in the reduction process according to the mechanism given in fig. 2. The proposed method was successfully applied to the determination of the trazodone in pure form and in formulations and the biological fluids (human urine and plasma).

A review of the electrochemical methods for the determination of some psychotropic drugs (e.g. phenothiazines, azaphenothiazines, dibenzoazepines, thioxanthenes)[68,72–89] is presented in Table 3 and the electrochemical methods for the determination of new atypical psychotropic drugs (e.g. olanzapine, sertraline, trazodone)[90–92] are given in Table 4.

**TABLE 3:** VOLTAMMETRIC ANALYSIS OF PSYCHOTROPIC DRUGS

Psychotropic drug	Medium	Method	Working electrode	Range of determination [mol/l]	LOD [mol/l]	Practical application	Ref.
Chlorpromazine,	Britton's buffer, pH=2-7	DPP	Hg	6·10^−6^ - 1·10^−4^	3·10^−7^	Drug	58
Promazine,				5·10^−6^ - 8·10^−5^	3·10^−7^	preparations	
Promethazine				8·10^−6^ - 1·10^−4^	4·10^−7^	Urine	
Chlorpromazine	0.2 M H_2_SO_4_	LSV	Ru	2·10^−4^ - 8·10^−4^	-	Drugs preparations	72
Thioridazine	0.2 M H_2_SO_4_	CV	Pt,	5·10^−5^ - 1·10^−3^	-	Drugs preparations	73
			Ru,	6·10^−4^ - 1·10^−2^			
			GC	1·10^−4^ - 1·10^−3^			
Fluphenazine	0.5 M H_2_SO_4_,	CV	Pt,	4·10^−4^ - 1·10^−2^	-	Drugs preparations	74
	Phosphate buffer, pH=6.2			2·10^−4^ - 4·10^−3^			
	0.5 M H_2_SO_4_		GC	2·10^−5^ - 8·10^−4^		
Promazine,	0.1 M HCl	LSV	CPE,	2.5·10^−5^- 5·10^−3^	-	Drugs preparations	75
Promethazine,			SCPE	2.5·10^−5^- 5·10^−3^		
Levomepromazine			GC	6.2·10^−5^-1.2·10^−3^		
Chlorpromazine,	0.1 M NaClO_4_	DPV	Pt	7·10^−7^ -1.4·10^−5^	4·10^−7^	Drugs preparations	76
Thioridazine	in acetonitrile						
Chlorpromazine,	Britton's buffer, pH=7.0	AdSV	Hg	2·10^−8^ -5·10^−6^	4.2·10^−9^	Drugs preparations	62
	(in the presence of Triton X-100)					Urine	
Ethopropazine	0.05 M Phthalate buffer, pH=3.5 (in the presence of SDS)	CV	Au	4·10^−7^ - 4·10^−6^	-	-	77
Promethiazine, Diethazine, Trifluoperazine, Fluphenazine	Phosphate buffer, pH=7	DPV	WIGE	5·10^−7^ - 1·10^−4^	5·10^−8^	Urine, plasma	63
Chlorpromazine	Phosphate buffer, pH=7	DPV	WIGE	4.8·10^−8^ - 2.4·10^−4^	5·10^−9^	Urine	78
Chlorpromazine,	0.1 M Phosphate	AdSV	CPE	8.3·10^−8^ - 2·10^−6^	1·10^−9^	Urine,	79,
Perphenazine, Promazine	buffer, pH=7.4	DPV				blood samples	80
Chlorpromazine	0.1 M Phosphate	AdSV	CPE	1.5·10^−6^ - 9·10^−6^	1·10^−7^	Urine	81
	buffer, pH=7.4	DPV			
Chlorpromazine,	Britton's buffer, pH=9	AdSV	GC	1.5·10^−7^ - 3.4·10^−6^	1.3·10^−7^	Blood	82
Promethiazine		DPV		3·10^−7^ - 3·10^−6^	1.2·10^−7^	samples	
Phenothiazine,	Acetate buffer,	CV	MCPE	2·10^−8^ - 3·10^−7^	1.2·10^−8^	Drugs'	83
Chlorpromazine,	pH=5.0				7·10^−9^	preparations	
promethiazine					5·10^−9^		
Thioridazine,	Acetate buffer,	DPV	MCPE	1·10^−7^ - 1·10^−6^	4.5·10^−8^	Drugs'	84
Prochlorperazine, Chlorpromazine	pH=5				1.2·10^−8^	preparations	
Chlorpromazine,	Phosphate	DPV	MCPE	1.96·10^−7^ -	1·10^−7^	Model	64
Thioridazine,	buffer, pH=7.4			2.75·10^−6^	7·10^−8^	serum	
Prochlorperazine,					4·10^−8^		
Levomepromazine					8·10^−8^		
Thioridazine	Phosphate	AdSV	MCPE	1·10^−8^ - 1·10^−7^	7·10^−9^	Drugs'	65
	buffer, pH=6.6	CV, DPV				preparations	
Fluphenazine	0.05 M HCOOH-	CV	MAu	5·10^−8^ - 1.5·10^−5^	1·10^−8^	Drugs'	85
	HCOONa buffer, pH=3.5						preparations
Perphenazine	0.05 M borate	CV	MAu	6·10^−9^ - 5·10^−7^		Drugs'	86
	buffer, pH=10.0			5·10^−7^ - 5·10^−6^	-	preparations	
Chlorpromazine	0.05 M Phosphate	CV	MAu	6·10^−6^ - 5·10^−5^	-	Biological	61
	buffer, pH=9					fluids	
Promazine,	Acetate buffer, pH=4.7	CV,	CPE,	2·10^−7^ - 3·10^−5^	down to 1·10^−8^	Drugs'	87
Promethiazine, Trifluoperazine, Chlorpromazine, Thioridazine		A	SCPE			preparations
Prothipendyl	Britton's buffer,	DPP	Hg	-	-	Blood, plasma,	59
	pH=3.5					urine
Imipramine	0.1 M H_2_SO_4_,	CV	MCPE	6·10^−5^ - 8·10^−4^	-	Drugs'	60
	Phosphate buffer, pH=7.4					preparations	
Imipramine,	Phosphate buffer,	AdSV	MCPE	1·10^−7^ - 1·10^−6^	2·10^−8^	Drugs'	65
Trimipramine	pH=6.6	CV, DPV				preparations
Imipramine,	Phosphate buffer,	DPV	MCPE	1.96·10^−7^ - 2.4·10^−6^	1·10^−7^	Plasma	64
Trimipramine,	pH=7.4				1.1·10^−7^		
Clomipramine					0.5·10^−7^			
Imipramine	Phosphate buffer,	LSV	MCPE	1·10^−7^ - 8·10^−6^	-	Drugs'	88
	pH=9					preparations, urine	
Imipramine,	Acetate buffer,	DPV	MCPE	1·10^−7^ - 1·10^−6^	8.5 ·10^−8^	Drugs'	84
Clomipramine,	pH=5				9.2 ·10^−8^	preparations	
Trimipramine					9.0 ·10^−8^		
Imipramine,	Phosphate buffer,	CV,	CPE,	2·10^−7^ - 6·10^−7^	1.5·10^−8^	Urine	67
Desipramine,	pH=9	DPV	GC	2·10^−7^ - 6·10^−7^	1.7·10^−8^		
Trimipramine				2·10^−7^ - 1.6·10^−6^	1.4·10^−8^		
Clomipramine	Britton's buffer, phosphate buffer	SWP	Hg	-	-	Drugs' preparations	89
Imipramine,	Phosphate buffer,	CV	GC,	-	-	Plasma	6
Clomipramine, Dezipramine	pH=6.9		BDD			
Imipramine,	0.1 H_2_SO_4_	LSV,	Pt,	-	-	-	57
Clomipramine, Dezipramine, Trimipramine		CV	Au				
Zuclopenthixol	0.1 M H_2_SO_4_,	CV,	GC	8·10^−7^ - 2·10^−4^	2.2·10^−7^	Drugs' preparations	68
	Britton's buffer, pH=2.0 - 11.5, phosphate buffer, pH=5.2 - 8.3	LSV, DPV							
Chlorprothixene	Britton's buffer, pH=8.2	CV, DPV	GC	0.1 - 1 μg/ml time of condition =30s 0.01 - 1 μg/ml time of condition = 120 s	-	Urine	69
Chlorprothixene, Thiothixene	0.1 M HCl, Britton's buffer, pH=10.13	dc-P	Hg	2.7·10^−5^ - 1·10^−4^	-	Drugs' preparations	70

AdSV=adsorptive stripping voltammetry, CV= cyclic voltammetry, DPV= differential pulse voltammetry, LSV= linear sweep voltammetry, SWP = square wave polarography, dc-P=direct current polarography, ac-P=alternating current polarography, DPP=differential pulse polarography, A=amperometry, CPE= carbon paste electrode, SCPE= solid carbon paste electrode, GC=glassy carbon electrode, MCPE= modified carbon paste electrode, WIGE= wax-impregnated graphite electrode, BDD= boron-doped diamond, Ru= ruthenium electrode, Pt= platinum electrode, Au= gold electrode, MAu= modified gold electrode, RDE= rotating disc electrode, SDS= sodium dodecylsulfate.

**TABLE 4:** VOLTAMMETRIC ANALYSIS OF ATYPICAL PSYCHOTROPIC DRUGS

Psychotropic drug	Medium	Method	Working electrode	Range of determination [mol/l]	LOD [mol/l]	Practical application	Ref.
Olanzapine	Phosphate buffer, pH=2.5	LSV	GC	1.97·10^−5^-1.59·10^−4^	9.54·10^−6^	Drugs preparations	90
Fluoxetine	Ringer buffer, pH=12	AdSV	Hg			-	91
		CV,		-	-	
		DPV		-	-			
		SWV		5.2·10^−5^ -5.2·10^−5^	3.9·10^−8^	
Paroxetine	Borate buffer, pH=8.8	AdSV, SWV,	Hg	3·10^−6^ - 1.7·10^−5^	4.8·10^−7^	Drugs preparations	17
Trazodone	Britton's buffer,	ac-P,	Hg	-	-	Urine, plasma	71
	pH=10	dc-P, DPV		9.8·10^−6^ -7.8·10^−5^	7.69·10^−7^		
				1.9·10^−6^ -5.8·10^−5^	2.54·10^−7^		
Sertraline	Borate buffer,	FIA	Hg	2·10^−7^ -1.2·10^−6^	1.5·10^−7^	Drugs preparations	18
	pH=8.2	AdSV					
		SWV				
Sertraline	Borate buffer, pH=8.2	AdSV SWV	Hg	2.33·10^−7^ - 3.15·10^−6^	1.98·10^−7^	Drugs preparations	92

## CONCLUSIONS

Psychotropic drugs, *e.g.* dibenzoazepines, dibenzocycloheptadienes, thioxanthenes, and phenothiazines, and new generation drugs, *e.g.* fluoxetine, fluvoxamine, and trazodone form cations which react with some organic substances (picric acid, flavianic acid, alizarin S, brilliant blue, and triphenylmethane dyes) and thiocyanate or halide anionic complexes (e.g. Co(II), Pd(II), Fe(III), Cr(III), Au(III), Ti(IV), Pt(IV), Mo(V), W(V), U(VI) forming ion-association compounds. The compounds get precipitated from aqueous solutions and can be quantitatively extracted into organic solvents (e.g. chloroform, dichloromethane and butanol). The extracts are intensely colored and stable for 1-3 days. These properties have been applied for the determination of above - mentioned metal ions and active compounds in pharmaceutical preparations.

As mentioned previously, the official compendia[93] recommends determination of psychotropic active substances in bulk or in pharmaceutical forms by measurement of the absorbance at selected wavelengths, or titration in a non–aqueous medium with potentiometric or visual indication at the end-point. The proposed pharmacopoeial procedures require intensive isolation and purification steps in the case of the assay of the studied psychotropic substances in their pharmaceutical dosage forms. The main disadvantage of direct UV spectrophotometry is the sensitivity to excipients usually presented in pharmaceutical formulations.

In the presented review, methods based on the complexation reactions are discussed as alternative methods. The absorbance of colored ion association complexes of psychotropic drugs are less liable to spectral interferences from other ingredients of pharmaceuticals. The reviewed methods offer advantages of their simplicity, rapidity and common access to instrumentation. The analytical methods for the determination of psychotropic drugs are characterized with good precision, sensitivity and reproducibility.

Electrochemical methods have also been used for the study and determination of some psychotropic drugs. Among the methods, mainly used are voltammetry - cyclic voltammetry and differential pulse voltammetry, after pre-concentration of studied substances at several kinds of bare surfaces of electrodes, e.g. glassy carbon electrode, carbon paste electrodes or using different modified electrodes. Procedures have been described in literature for the determination of dibenzoazepines, dibenzocycloheptadienes, thioxanthenes, phenothiazines and new generation psychotropic drugs such as olanzapine, fluoxetine, paroxetine, trazodone, sertraline. Those are projected to be simple, fast and sensitive, which can be applied successfully to determine active substances, their metabolites in pharmaceutical formulations and biological fluids.
